# First report of the family Ideoroncidae (Arachnida, Pseudoscorpiones) from China, with description of a new species

**DOI:** 10.3897/BDJ.12.e117319

**Published:** 2024-03-20

**Authors:** Zhizhong Gao, Kaiquan Zhang, Feng Zhang

**Affiliations:** 1 Department of Biology, Xinzhou Normal University, Xinzhou, China Department of Biology, Xinzhou Normal University Xinzhou China; 2 The Key Laboratory of Zoological Systematics and Application, Institute of Life Science and Green Development, College of Life Sciences, Hebei University, Baoding, China The Key Laboratory of Zoological Systematics and Application, Institute of Life Science and Green Development, College of Life Sciences, Hebei University Baoding China

**Keywords:** taxonomy, pseudoscorpion, China

## Abstract

**Background:**

The pseudoscorpion genus *Shravana* Chamberlin, 1930, belonging to the family Ideoroncidae Chamberlin, 1930, currently contains 13 species. To date, no ideoroncid species has been recorded from China.

**New information:**

The family Ideoroncidae Chamberlin, 1930 is reported from China for the first time. A new species of the genus *Shravana* Chamberlin, 1930, collected from Xizang Autonomous Region, China, is described under the name of *Shravanazhui* Gao & Zhang, sp. nov. on the basis of both sexes. The finding of this new species expands the distribution range of this family in Asia.

## Introduction

The pseudoscorpion family Ideoroncidae was first recognised by Chamberlin (1930) and is discontinuously distributed around the world. To date, the family currently includes 84 modern species in 14 recognised genera, distributed in the Americas, Africa and Asia. Amongst them, three occur in south-eastern Asia: *Dhanus* Chamberlin, 1930, *Shravana* Chamberlin, 1930 and *Sironcus* Harvey, 2016 (Harvey and Muchmore 2013, Harvey and Gerhard 2014, Harvey 2016, WPC 2022).

The genus *Shravana* was erected by Chamberlin (1930), It contains 13 species, which are distributed sporadically throughout Asia and the Socotran Archipelago (*Harvey 2016, WPC 2022*).

Due to a lack of comprehensive fieldwork, up to the present, no *Shravana* species has been reported from China. *Shravanazhui* sp. nov. is presented here as a representative of the family Ideoroncidae recorded in China for the first time.

## Materials and methods

The specimens are preserved in 75% alcohol and deposited in the Museum of Hebei University (**MHBU**) (Baoding, China). Photographs were taken with a Leica M205a stereomicroscope, equipped with a Leica DFC550 camera and the LAS software v. 4.6 and the Leica M205A stereomicroscope with a drawing tube was used for drawings and measurements. All measurements are given in mm unless noted otherwise. The chela and the chelal hand were measured in the ventral view. Detailed examination was carried out with an Olympus BX53 general optical microscope. Temporary slide mounts were made in glycerol.

Terminology and mensuration largely follow Chamberlin (1931), except for the nomenclature of the pedipalps, legs and with some minor modifications to the terminology of the trichobothria (Harvey 1992), chelicera (Harvey and Edward 2007, Judson 2007) and faces of the appendages (Harvey et al. 2012). The notation of the supernumerary trichobothria follows Mahnert (1984).

## Taxon treatments

### Shravana zhui

Gao & Zhang sp. nov.

6AA594E5-8698-50E1-8429-AEF99F20532D

7C2CE98C-5E08-43DD-87FB-DB4EB60FD4C7

#### Materials

- **Type status:** Holotype. **Occurrence:** individualCount: 1; sex: male; lifeStage: adults; occurrenceID: D99B349E-2A03-5F69-856D-E3B8D8039759; **Taxon:** scientificName: *Shravanazhui*; family: Ideoroncidae; **Location:** country: China; stateProvince: Xizang Autonomous Region; county: Lang County; locality: Dongga Town; verbatimElevation: 3062 m; verbatimLatitude: 29°05.053′N; verbatimLongitude: 93°08.995′E; **Event:** year: 2014; month: 8; day: 11; **Record Level:** institutionID: the Museum of Hebei University; institutionCode: MHBU; collectionCode: Ps.-MHBU- XZ14081101
- **Type status:** Paratype. **Occurrence:** individualCount: 1; sex: female; lifeStage: adult; occurrenceID: D87124B9-C664-52E6-B0AE-B38AE570E2B1; **Taxon:** scientificName: *Shravanazhui*; family: Ideoroncidae; **Location:** country: China; stateProvince: Xizang Autonomous Region; county: Lang County; locality: Dongga Town; verbatimElevation: 3062 m; verbatimLatitude: 29°05.053′N; verbatimLongitude: 93°08.995′E; **Event:** year: 2014; month: 8; day: 11; **Record Level:** institutionID: the Museum of Hebei University; institutionCode: MHBU; collectionCode: Ps.-MHBU- XZ14081102

#### Description

**Figure 1.**
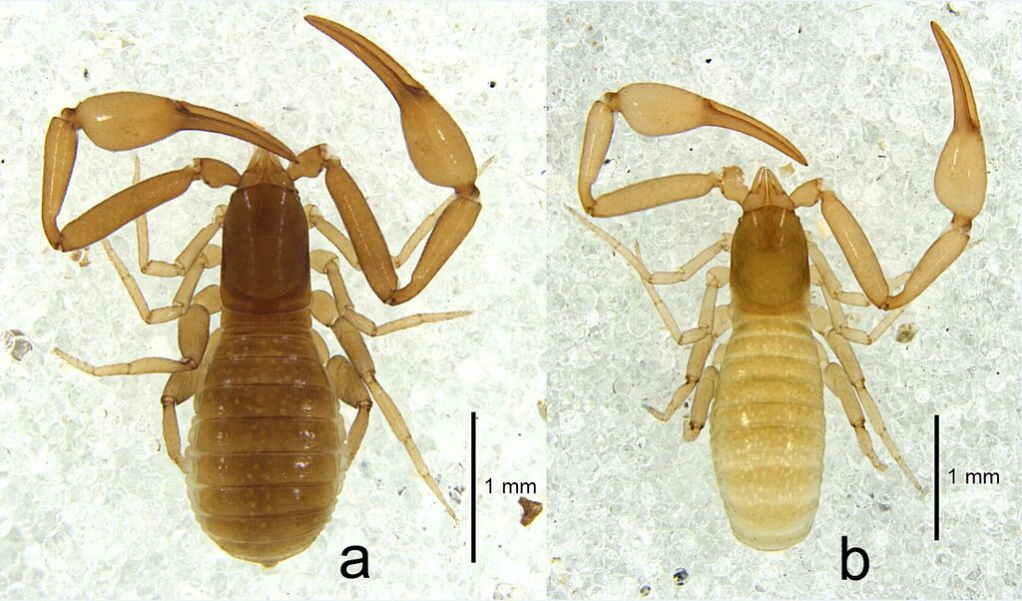
***Shravanazhui*** **sp. nov.**, a. holotype, dorsal view; b. female paratype, dorsal view.

**Adult male.** Carapace and tergites dark brown, chelicerae, pedipalps and legs yellowish-brown (Fig. 1a). Colour of females lighter than males.

Setae: generally long, straight or slightly curved and acicular.

**Figure 2.**
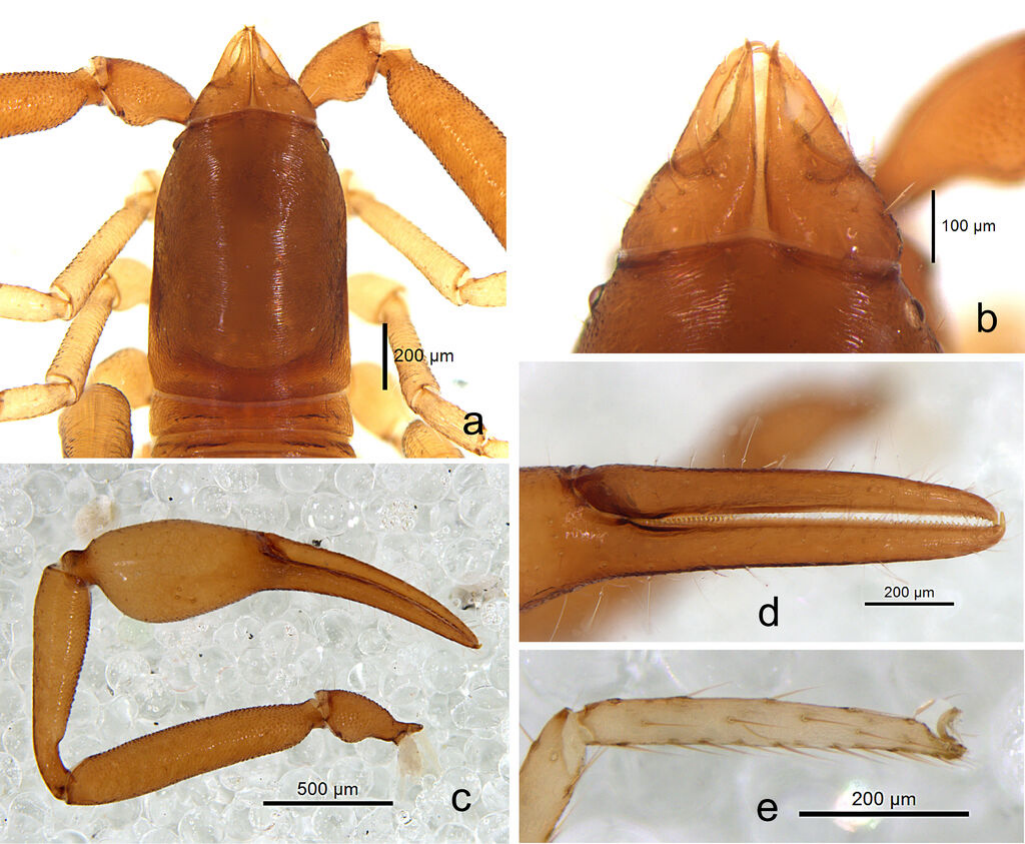
*Shravanazhui* sp. nov., holotype: **a** carapace, dorsal view; **b** chelicera, dorsal view; **c** pedipalp, dorsal view; **d** chelal fingers, prolateral view; **e** tarsus of leg IV, lateral view.

**Figure 3.**
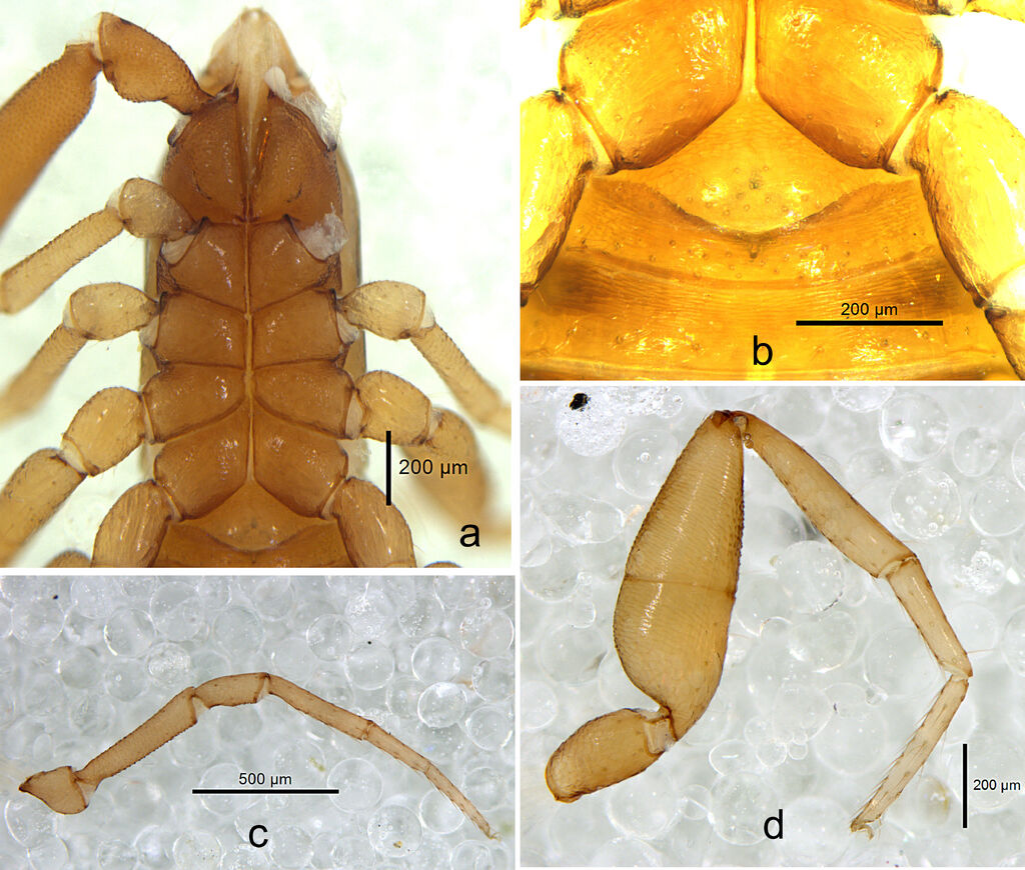
*Shravanazhui* sp. nov., holotype: **a** coxae, ventral view; **b** genital area, ventral view; **c** Leg I, lateral view; **d** Leg IV, lateral view.

**Figure 4.**
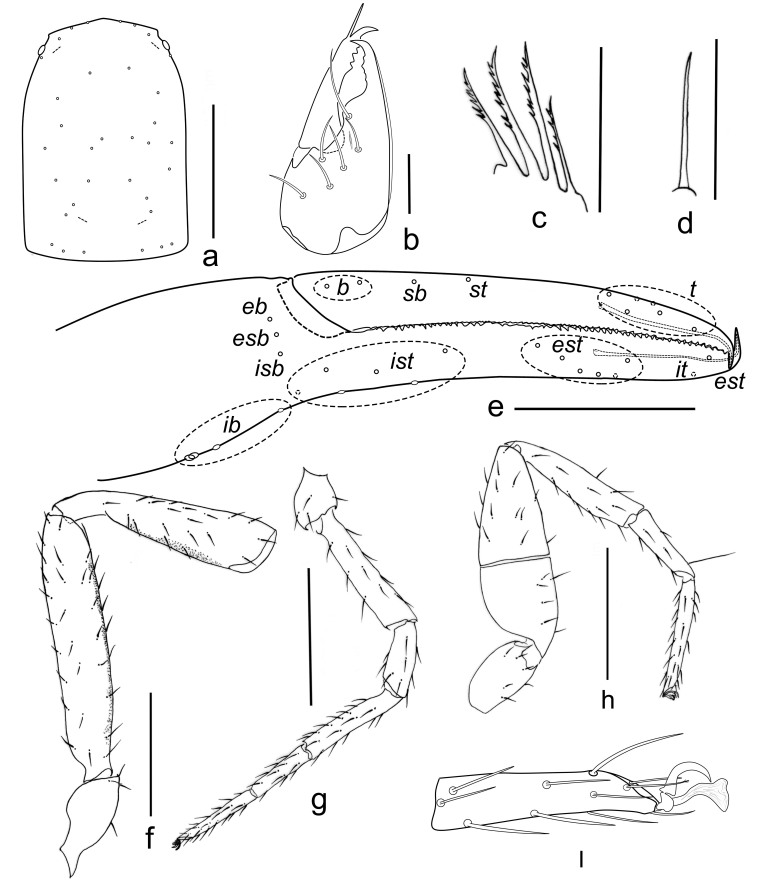
*Shravanazhui* sp. nov., holotype: **a** carapace (dorsal view); **b** left chelicera (dorsal view); **c** rallum; **d** galea; **e** chelal fingers (prolateral view); **f** left palp (minus chela, dorsal view); **g** leg I (lateral view); **h** leg IV; **i** tarsus IV (lateral view). Scale bars: 0.10 mm (b–d); 0.50 mm (a, e–h).

**Cephalothorax** (Figs 2, 4). Carapace (Fig. 2a, Fig. 4a) distinctly longer than broad (1.50 times); 2 bulging eyes; surface of carapace broadly reticulate; blunt rounded epistome present on anterior margin; 34 setae on the carapace, include 4 setae near anterior and 6 near posterior margin, with shallow posterior furrow (same in the females); four lyrifissures present (marked as dotted lines). **Coxal region** (Fig. 3a). Manducatory process with 2 long apical setae, chelal coxa with 6–8 setae (1 tactile seta), chaetotaxy of coxa I 7, II 5, III 7, IV 6. **Chelicera** (Fig. 2b, Fig. 4b) palm with 6 simple setae; fixed finger with about 5 small teeth, movable finger with 6 erect and pointed teeth; galea (Fig. 4d) simple, smooth, long and straight; serrula exterior with 18–19 blades, serrula interior with 14–16 blades, thin lamina exterior present. Rallum (Fig. 4c) in a row and composed of 4 anteriorly dentate blades. **Pedipalps** (Fig. 2c–d, Fig. 4e–f): Trochanter, femur and patella medially coarsely granulate (Fig. 2c), hand medio-distally finely granulate; trochanter with a small round dorsal hump, 2.11 times longer than broad, femur 4.73 times longer than broad, patella 4.05 times longer than broad, fixed finger with 38–44 acute teeth (females with 39–42), which, in the middle, smaller than that in both ends; movable finger with 36–42 irregular acute teeth (females with 37–40); 31 trichobothria (21+10), 3 on prolateral hand face. Venom apparatus present in both chelal fingers, venom duct long, see Fig. 4e. **Opisthosoma.** Abdomen Ovate; tergites and sternites undivided and uniseriate, all setae acuminate; tergites brown-yellowish, sternites yellowish; tergal chaetotaxy: 4: 6: 8: 9: 9: 8: 10: 9: 9: 8: 5, including 2 tactile setae on the last tergites; white spot on each lateral side of sternites V-IX, sternal chaetotaxy (IV–XI): 10: 11: 10: 12: 10: 10: 10: 7, including 2 tactile setae on the last sternites; anterior genital operculum with a median and posterior group of 10–12 setae (Fig. 3b); posterior genital sternite with 16–17 setae (Fig. 3b); pleural membrane finely striate. **Legs.** Leg I (Fig. 2e, Fig. 3c, Fig. 4g) slender, trochanter, femur and patella medially coarsely granulate. Leg IV (Fig. 3d, Fig. 4h) stout, with one tactile seta present on basitarsus (TS = 0.21), subterminal tarsal seta apically trifurcate, arolium undivided, without ventral hooked process, slightly longer than the smooth and simple claws (Fig. 2e, Fig. 4i). **Measurements (length/breadth or depth in mm, ratios in parentheses).** Male (holotype). Body length 2.53. Carapace 0.87 × 0.58 (1.50). Palpal trochanter 0.40 × 0.19 (2.11), femur 1.04 × 0.22 (4.73), patella 0.89 × 0.22 (4.05), chela (with pedicel) 1.67/0.40, chela (without pedicel) 1.58/0.40, hand length (with pedicel) 0.77, hand length (without pedicel) 0.67, movable finger length 0.96. Leg I trochanter 0.22 × 0.16 (1.38), femur 0.51 × 0.11 (4.64), patella 0.26 × 0.11 (2.36), tibia 0.41 × 0.08 (5.13), basitarsus 0.25/0.06 (4.17), telotarsus 0.32/0.05 (6.40). Leg IV trochanter 0.30 × 0.16 (1.88), femur+patella 0.79 × 0.27 (2.93), tibia 0.56 × 0.12 (4.67), basitarsus 0.31/0.09 (3.44), telotarsus 0.42/0.06 (7.00). **Measurements (female paratype).** Mostly same as holotype (Fig. 1b), body length 2.67. Carapace 0.88 × 0.61 (1.44). Palpal trochanter 0.38×0.19 (2.00), femur 1.08 × 0.23 (4.70), patella 0.92 × 0.22 (4.18), chela (with pedicel) 1.67 × 0.40 (4.18), chela (without pedicel) 1.58 × 0.40 (3.95), hand length (with pedicel) 0.81, hand length (without pedicel) 0.70, movable ﬁnger length 1.04. Leg I trochanter 0.24 × 0.16 (1.50), femur 0.52 × 0.12 (4.33), patella 0.27 × 0.12 (2.25), tibia 0.42 × 0.07 (6.00), basitarsus 0.28/0.06 (4.67), telotarsus 0.36/0.05 (7.20); leg IV trochanter 0.32 × 0.18 (1.78), femur+patella 0.80 × 0.26 (3.08), tibia 0.56 × 0.12 (4.67), basitarsus 0.36/0.09 (4.00), telotarsus 0.45/0.06 (7.50).

##### Comparison

The new species share the character of presence of only two trichobothria in the *b* region with *S.pohli* (Mahnert, 2007) and *S.socotraensis* (Mahnert, 2007); it differs from them by the presence of 21 trichobothria on the fixed chelal finger and hand, whereas *S.pohli* has 23–24, *S.socotraensis* has only 20 trichobothria (Mahnert 2007, Harvey 2016)

#### Diagnosis

For the diagnosis for the genus *Shravana*, see Harvey (2016). This new species differs from all the other members of the genus *Shravana* by the following combination of characteristics: thin lamina exterior present, arolia without a ventral hook and slightly longer than the claws; 21 trichobothria on chelal fixed fingers, 10 on movable fingers, two trichobothria in the *b* region of the chela; tergites and sternites undivided, white spot on each lateral side of sternites V-IX; the presence of 34 setae on the carapace, include four near anterior margin, six near the posterior margin, with shallow posterior furrow; slender chela (femur 4.73 times, patella 4.05 times longer than broad); cheliceral palm with six simple setae.

#### Etymology

This species is named in memory of Prof. Mingsheng Zhu (1950-2010), a famous arachnologist from China, who made a great contribution to the study of Chinese arachnology.

#### Distribution

The new species is known only from Xizang Autonomous Region, China.

#### Notes

*Shravanazhui* sp. nov. is the first member of the family Ideoroncidae to be discovered in China, which expanded the distribution range of this family in Asia; two specimens were collected on the wet inner side under stones at a relatively higher elevation (3062 m).

## Supplementary Material

XML Treatment for Shravana zhui
